# Longitudinal mouse-PET imaging: a reliable method for estimating binding parameters without a reference region or blood sampling

**DOI:** 10.1007/s00259-020-04755-5

**Published:** 2020-03-24

**Authors:** Catriona Wimberley, Duc Loc Nguyen, Charles Truillet, Marie-Anne Peyronneau, Zuhal Gulhan, Matteo Tonietto, Fawzi Boumezbeur, Raphael Boisgard, Sylvie Chalon, Viviane Bouilleret, Irène Buvat

**Affiliations:** 1 Laboratoire d’Imagerie Moléculaire In Vivo (IMIV), Université Paris-Saclay, Inserm, CNRS, CEA, Orsay, France; 2 Edinburgh Imaging Facility, QMRI, The University of Edinburgh, Chancellor’s building, 49 Little France Crescent, BioQuarter, Edinburgh, EH164SB UK; 3 UMR 1253, iBrain, Inserm, Université de Tours, Tours, France; 4 Institut du Cerveau et de la Moelle épinière (ICM), Hôpital de la Pitié Salpêtrière, Paris, France; 5 NeuroSpin, CEA, CNRS, Université Paris-Saclay, Gif-sur-Yvette, France; 6 Neurophysiology and Epileptology, Université Paris Saclay-APHP, Le Kremlin-Bicêtre, France; 7 Laboratoire d’Imagerie Translationnelle en Oncologie (LITO), Inserm, Institut Curie, Orsay, France

**Keywords:** PET, TSPO, Factor analysis, Mouse, Image-derived input function

## Abstract

**Abstract:**

Longitudinal mouse PET imaging is becoming increasingly popular due to the large number of transgenic and disease models available but faces challenges. These challenges are related to the small size of the mouse brain and the limited spatial resolution of microPET scanners, along with the small blood volume making arterial blood sampling challenging and impossible for longitudinal studies. The ability to extract an input function directly from the image would be useful for quantification in longitudinal small animal studies where there is no true reference region available such as TSPO imaging.

**Methods:**

Using dynamic, whole-body ^18^F-DPA-714 PET scans (60 min) in a mouse model of hippocampal sclerosis, we applied a factor analysis (FA) approach to extract an image-derived input function (IDIF). This mouse-specific IDIF was then used for 4D-resolution recovery and denoising (4D-RRD) that outputs a dynamic image with better spatial resolution and noise properties, and a map of the total volume of distribution (V_T_) was obtained using a basis function approach in a total of 9 mice with 4 longitudinal PET scans each. We also calculated percent injected dose (%ID) with and without 4D-RRD. The V_T_ and %ID parameters were compared to quantified ex vivo autoradiography using regional correlations of the specific binding from autoradiography against V_T_ and %ID parameters.

**Results:**

The peaks of the IDIFs were strongly correlated with the injected dose (Pearson *R* = 0.79). The regional correlations between the %ID estimates and autoradiography were *R* = 0.53 without 4D-RRD and 0.72 with 4D-RRD over all mice and scans. The regional correlations between the V_T_ estimates and autoradiography were *R* = 0.66 without 4D-RRD and 0.79 with application of 4D-RRD over all mice and scans.

**Conclusion:**

We present a FA approach for IDIF extraction which is robust, reproducible and can be used in quantification methods for resolution recovery, denoising and parameter estimation. We demonstrated that the proposed quantification method yields parameter estimates closer to ex vivo measurements than semi-quantitative methods such as %ID and is immune to tracer binding in tissue unlike reference tissue methods. This approach allows for accurate quantification in longitudinal PET studies in mice while avoiding repeated blood sampling.

**Electronic supplementary material:**

The online version of this article (10.1007/s00259-020-04755-5) contains supplementary material, which is available to authorized users.

## Background

Dedicated PET machines enable mouse imaging with the large number of transgenic mice and disease models available [1]. In longitudinal PET studies, the changes in receptor or protein expression can be small, which means that sophisticated quantification and physiological parameter estimation methods would be needed to identify such small variations. The gold standard parameter estimation method is to use compartmental analysis with a metabolite-corrected plasma curve from arterial blood sampling [2]. However, blood sampling throughout a mouse PET scan is extremely difficult and impossible in a longitudinal study due to the limited blood volume of the mouse.

For some targets, there is the possibility to use a reference region for compartmental modelling to estimate binding parameters. This is not the case for targets where there is no brain region without the target present, such as the translocator protein (TSPO). TSPO has been demonstrated as a biomarker in diseases that have a component of neuroinflammation such as epilepsy and Alzheimer’s disease. Among the most commonly used TSPO tracers, ^18^F-DPA-714 was recently shown to be able to follow TSPO expression longitudinally in a mouse model of epilepsy with hippocampal sclerosis [3, 4]. Due to the low-level basal expression of TSPO in healthy brain tissue, and because the pattern of over-expression is unknown in some disease states, using a reference region is suboptimal.

Additionally, the small size of the mouse brain compared to the limited spatial resolution of the PET scanner induces significant partial volume effects between brain structures that need to be corrected for accurate activity quantification and subsequent parameter estimation in brain studies [5, 6].

Current methods of quantification for mouse TSPO PET studies have been relatively simple and include measurements of standardized uptake values (SUV) or percent injected dose per cc (%ID) [3, 4]. For some studies where the longitudinal pattern of pathology is known, an anatomical pseudo-reference region was used [7, 8]. Another group performed a normalization against a manually defined region of interest (ROI) over cardiac tissue [9]. Most studies do not apply any partial volume effect (PVE) correction to the images further complicating the detection of group differences [5, 6].

We propose a generic method for quantification of TSPO PET scans in the mouse by taking the focus away from finding an appropriate reference or pseudo-reference region and using a data-driven approach instead, to extract a blood input function from the whole-body image of the mouse.

To obtain an image-derived input function (IDIF) for mouse scans, a common method is to define a ROI over the aorta or a blood pool in the heart [10, 11]. This is not always ideal due to the limited spatial resolution producing strong PVE in the small vessel and cardiac structures, resulting in a highly contaminated blood curve. To overcome these limitations, one group tested a PVE correction that uses anatomical information from an MRI [12]. Yet, for TSPO tracers, this method will not give an adequate blood curve due to the high level of TSPO expression in the heart [13] and blood vessels [14]. To avoid the need for manual VOI placement or precise image registration techniques, we investigated factor analysis (FA) for estimating the blood curve and estimating the TSPO-tracer binding signal.

FA approaches have already been used in PET imaging, for noise removal [15], for the separation of biological signals [16–20] and also for IDIF extraction in canines [21], humans [22, 23] and in the mouse [24]. However, using FA to derive an accurate IDIF for binding parameter estimation in mouse studies has not yet been reported.

In this study, we present and evaluate a FA approach for extracting a robust IDIF from mouse ^18^F-DPA-714 PET scans for biological parameter estimation in the brain. This IDIF was used in a 4D-resolution recovery and noise reduction algorithm (4D-RRD) which performs iterative deconvolution combined with a basis function noise removal and enables subsequent parameter estimation [5, 6, 25]. The 4D-RRD process results in an image with higher spatial resolution and better noise properties than the original and produces a parametric map of the total volume of distribution (V_T_). The V_T_ maps were compared to quantitative ex vivo autoradiography using the structural analogue ^3^H-DPA-714. We also calculated the %ID for each scan, a commonly used semi-quantitative measure for comparison to the V_T_ and autoradiography to highlight the importance of using accurate and robust quantification methods.

## Methods

### Animal models

All experiments involving animals were conducted according to the European directive 2010/63/EU and its transposition in the French law (Décret n° 2013-118). Animal experiments were conducted at the imaging facility CEA-SHFJ (authorization D91-471-105/ethic committee n°44).

Experiments were performed on adult C57/BL6 male mice (4 months old) (Charles River, France) housed in individual cages. The mouse model of epilepsy was induced as previously described. Briefly, an injection of kainic acid (KA) was made into the right dorsal hippocampus [26, 27] of 21 mice (*n* = 9 for longitudinal PET imaging, *n* = 12 for ex vivo autoradiography).

### PET and MR imaging

Each mouse underwent ^18^F-DPA-714 scans before KA induction (baseline, *n* = 9) and after the KA induction at 7 days (*n* = 6), 1 month (*n* = 8) and 6 months (*n* = 8). Longitudinal scans were performed on the same animals whenever possible. Table 1 shows at which time point each mouse was scanned.

**Table 1:**
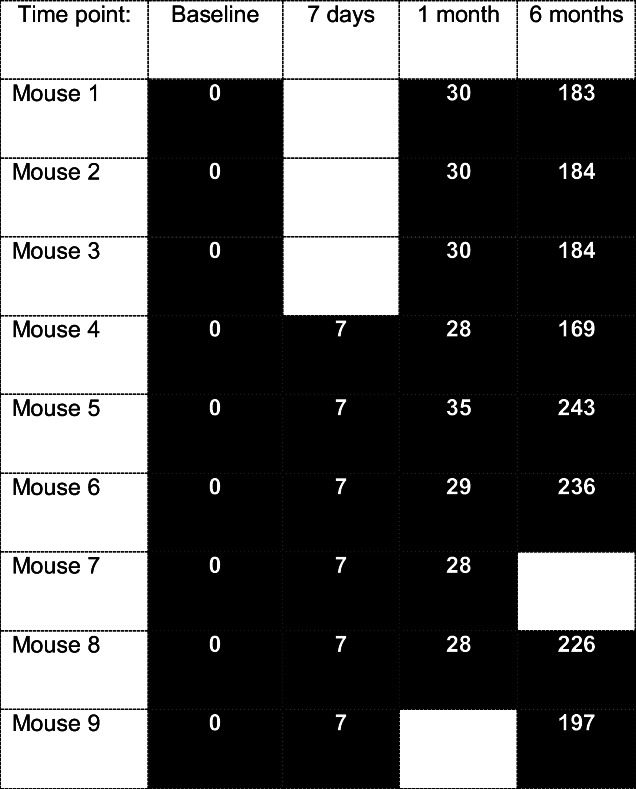
Outline of which mice had scans at which time point

The filled black table entry indicates that the mouse was scanned at that time point, and a white entry indicates the mouse did not undergo a scan. The number within each box is the number of days since KA induction

^18^F-DPA-714 was synthesized as previously described [28]. Mice were anesthetised with isoflurane (3.5% for induction, 1.5–2% for maintenance). Dynamic PET scans of 60 min (framing 3 × 30 seconds (s), 5 × 60 s, 5 × 120 s, 3 × 180 s, 3 × 240 s, 4 × 300 s, 1 × 240 s) were acquired with a tail vein injection (1 min using an injection pump) of 5.3 ± 1.6 MBq of ^18^F-DPA-714 (injected mass, 0.097 ± 0.04 nmol; specific activity, 66 ± 39 GBq/μmol; volume, 100 μl) using the Inveon microPET-CT (Siemens Medical Solutions, Knoxville, TN, USA). An additional cohort of healthy animals was used for a presaturation study (*n* = 6). These animals were given a large dose of non-radioactive DPA-714 before the radioactive tracer administration to saturate the TSPO binding (hot dose 0.33 ± 0.3 nmol, stable compound 64 ± 0.0 nmol). After the PET scan, a 6 min 80 kV/500 μA CT scan was performed for attenuation correction and for registering PET/CT images to an MRI template. Animals were scanned at the 6-month time point on an 11.7 T MRI scanner equipped with a CryoProbe dedicated for mouse brain imaging (Bruker BioSpin, Ettlingen, Germany) in order to acquire anatomical T_2_*-weighted MRI (Multi-gradient-echo sequence, TR = 100 ms, weighted average of 8 echo-images with TE ranging from 2.5 to 23.5 ms, resolution 80 × 80 × 160 μm) and assess morphological changes in the hippocampus and surrounding structures. Individual MRI was manually registered to an MRI atlas template [29].

### Image reconstruction

PET Images were reconstructed using a 2D OSEM iterative algorithm (4 iterations, 16 subsets, voxel size = 0.4 mm × 0.4 mm × 0.8 mm). Normalization, dead time correction, randoms subtraction, CT-based attenuation and scatter corrections were applied. In order to create average PET parametric maps for each of the time points, the PET scans were registered into the same space using the CT scans, which are acquired in the same reference space. The CT scans were cropped around the skull, and one baseline CT scan was chosen as reference which each of the others were registered to. For ROI definition of regions of interest, the reference CT was manually registered to an MRI atlas [29]. The transformation matrices between the PET, CT and MRI atlas were combined to position the ROI in the PET image volume.

### Autoradiography

The autoradiography was performed on four cohorts of mice (baseline, *n* = 3; 7 days, *n* = 3; 1 month, *n* = 3; and 6 months, *n* = 3) as described in Nguyen et al. [4]. The density of TSPO binding sites was measured by in vitro autoradiographic experiments using [^3^H]DPA714 (Specific Activity 2.01 GBq/μmol provided by F. Dollé, CEA, Institut des Sciences du Vivant Frédéric Joliot, SHFJ, Université Paris-Saclay, Orsay, France) according to the method used in Foucault-Fruchard et al. [30]. Non-specific binding was assessed in the presence of 1 μmol/L PK-11195 (Sigma-Aldrich, Saint-Quentin-Fallavier, France). ^3^H autoradiography was used because it provides better resolution and hence more accurate quantification compared to ^18^F [31].

For quantification, four sections were analysed per mouse. ROIs were manually drawn on seven regions (left and right hippocampus, cortex, thalamus and whole cerebellum) using the Paxinos atlas as reference [32]. These ROIs are presented in Supplementary Fig. 3. Using the β-vision software (Biospace Lab), the level of bound radioactivity was directly determined by counting the β-particles emitted from the delineated area. The radioligand signal in the ROIs was measured for each mouse and expressed as counts per minute per square millimetre (cpm/mm^2^). Specific binding was determined by subtracting non-specific binding (as described above) from total binding.

### Image-derived input function using factor analysis

FA was performed using PIXIES software (http://www.apteryx.fr/) on the original reconstructed dynamic scans. FA of medical images has been shown to effectively separate biological signals and remove noise [16–22, 33]. The FA model assumes that the dynamic image is made up of a limited number of fundamental spatial distributions which may or may not be overlapping, each one corresponding to a specific biological signal. The signal in each voxel from the original image over time (S_i_(t)) is expressed as a linear combination of factor curves (f_k_(t)) plus the error term (incorporating the noise and any modelling errors, e_i_(t)) [19]:

$$ {S}_i(t)={\sum}_{k=1}^K{a}_k(i){f}_k(t)+{e}_i(t). $$

The weights *a*_k_(i) correspond to the portion of signal *S*_i_(t) that follows the *f*_k_ time curve so that the *a*_k_ image reflects the spatial distribution of signal with the *f*_k_ kinetics.

**Fig. 1:**
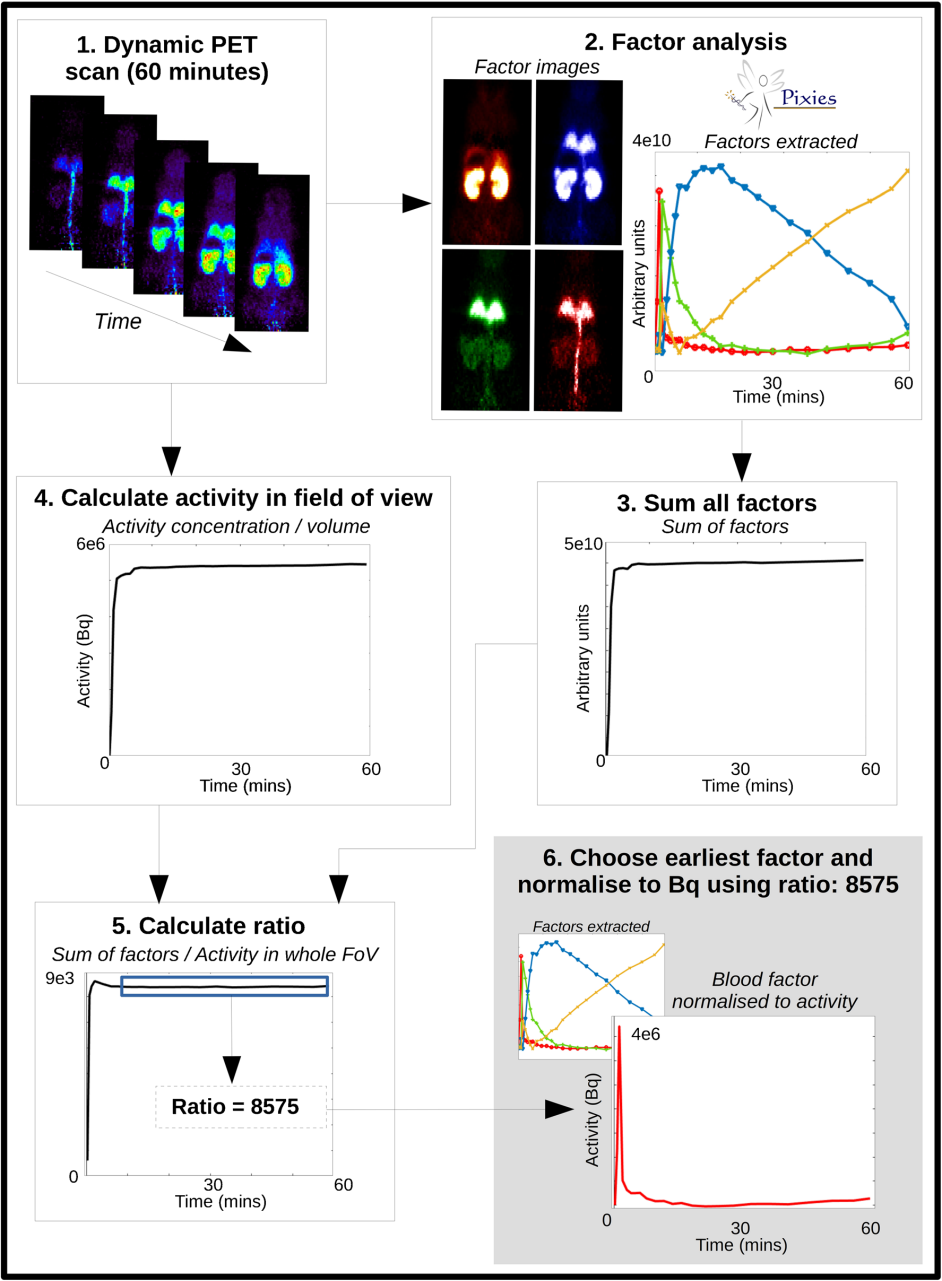
Process for extracting the IDIF from the dynamic PET scan: (1) the whole-body dynamic PET captures all activity; (2) FA is run with 3 (presaturation) or 4 (tracer dose) factors – the factor curves are shown along with their corresponding spatial distribution and relative intensity. The curve with the earliest peak (in red) shows the strongest signal in the expected regions for blood (tail vein, aorta, lungs); (3) all factors are summed together; (4) the whole-body TAC is obtained from a ROI placed around the whole body, and the total activity in Bq is calculated from the measured activity concentration (Bq/cc) multiplied by ROI volume; (5) the ratio between the summed factors and the whole-body TAC is calculated and the average of the ratio from 10 min onwards is calculated; (6) the blood factor curve is normalized to Bq using the average ratio value. This IDIF is then metabolite corrected and used in the image processing and parameter estimation

The FA model was solved in 2 steps. First, a principal component analysis (PCA) was performed, and the three 1st principal components were used to span the PCA space. The signal *S*_i_(t) in each voxel *i* was projected into that space, and a K-means algorithm was used to identify 4 clusters in that space. The centroids of these 4 clusters were used as initial factors *f*_k_(t). The identification of the final factors relies on a number of constraints reflecting priors [34]. We used the following constraints (Fig. 1) for all scans:

1. All factors and images should be positive.
2. One factor should have one maximum only with its maximum in the frame corresponding to 30–60 s.

Among the 4 factors, the one that best met P2 constraint was automatically identified as the IDIF factor. Then the factors were iteratively refined to meet P1 and P2 using the algorithm described in Benali et al. [34], with P2 applied to the previously identified IDIF factor. To force P2, the IDIF factor curve was altered when needed so as to have its maximum in the 30–60 s frame, to be increasing before that max and to be decreasing after, and the resulting curve was projected in the PCA space. The iterative process was automatically stopped when the factors did not change substantially between two successive iterations.

The factor curves were normalized to the activity within the scan (Bq) as shown in Fig. 1. The IDIF was then converted to %ID, and the mean and standard deviation over all curves obtained from all mice and all longitudinal PET scans in a given mouse were calculated.

### IDIF calculation: presaturation and tracer dose studies

TSPO present in the heart, lung, vascular tissue and in the blood can confound the estimation of the IDIF in tracer dose studies. When the TSPO is blocked with cold ligand in the presaturation experiments, the confounding binding component is removed from the total signal. For the presaturation scans, 3 factors were thus extracted, and priors P1 and P2 as above were used.

IDIF estimation from tracer dose scans was performed with one extra factor to account for the binding of the ligand and one extra constraint: P3. The shape of one factor should be similar to the average IDIF obtained from all 6 presaturation experiments.

The extracted IDIFs were metabolite corrected using a population metabolite curve (Supplementary Fig. 1) [35] and fitted using a previously described method [36].

### Image processing for spatial resolution recovery and noise removal

For spatial resolution recovery and noise removal, a 4D iterative deconvolution process is performed with a basis pursuit denoising for temporal regularization (4D-RRD) implemented in GNU Octave [5, 6, 37]. This method used a set of 12 basis functions generated by convolving an input function with a set of exponentials to fit the time-activity curve (TAC) from each voxel [25]. The input function used to generate the basis functions was the IDIF extracted from each individual animal which had been metabolite corrected. The output of 4D-RDD is a dynamic image that has higher spatial resolution and reduced noise compared to the original and a parametric map of the distribution volume, V_T_, which is estimated using the basis pursuit method as described in Gunn et al. 2002 [25]. For this study, the number of iterations used was 0, 10 and 15 iterations so that the results after each step of the image processing could be analysed. Zero iteration corresponds to the application of the denoising and V_T_ estimation only, with no partial volume correction.

### Parameters estimated

The parametric maps of V_T_ at 0, 10 and 15 iterations of 4D-RRD were used to calculate regional V_T_ values. The V_T_ values were calculated using the basis pursuit method (Gunn et al. 2005) which is integrated into the 4D-RDD resolution recovery and noise reduction method as described above [6]. The basis pursuit method of parameter estimation is particularly robust against noise and hence well suited for generating voxel-wise maps. The %ID was also calculated from the original images (without 4D-RDD) and from the images produced with 10 iterations of 4D-RRD. Regional values of V_T_ and %ID were extracted from the parametric images using the registered mouse brain atlas.

### Comparison of quantification method to autoradiography

The specific binding values were obtained from the autoradiography for the regions as described above. Regional group averages for the PET measures of %ID or V_T_ were correlated against the measured activity from the autoradiography slices for all animals at the different time points for 7 regions of interest (left and right cortex, hippocampus, thalamus and whole cerebellum). The autoradiography measurement represents only the specific binding of the tracer, whereas the V_T_ estimate represents the non-displaceable tracer (V_ND_ which incorporates the free tracer in tissue, V_F_, plus the non-specific binding, V_NS_) and the specific binding (V_S_). Ideally, V_T_ should have a direct relationship with specific binding, but may not, due to non-specific binding or differences in the free tracer in tissue.

## Results

### Factor analysis

Figure 1 (2) shows the four different factors identified in the mouse PET image of the tracer studies. The image beside each of the 4 identified factors in the corresponding colour shows the spatial distribution of the factor which can help to interpret the biological meaning of each curve. There is strong presence of the factor with the earliest peak (red) within the lungs, and the signal is very high in the abdominal artery/vein compared to the other factors, indicating that it is mainly coming from blood. There is little signal seen in the ventricles (not shown) for the blood factor, which could be due to the heart motion causing the signal to be more diluted than it is in the aorta. The factor that peaks second (green) appears in the lungs, kidneys and abdominal region could be related to free tracer in tissue, with a large proportion appearing in the lungs, which are highly perfused. The third factor in blue could describes the specific binding of the tracer, with high concentration in the kidneys and lungs, where it is known that there is high TSPO density, including in the mouse as shown in [13]. The factor in yellow shows a shape related to the accumulation of tracer and is strongest in the kidneys, which could therefore be related to excretion. There is very little of this signal seen in the lungs or the rest of the body.

### IDIF obtained using factor analysis

**Fig. 2:**
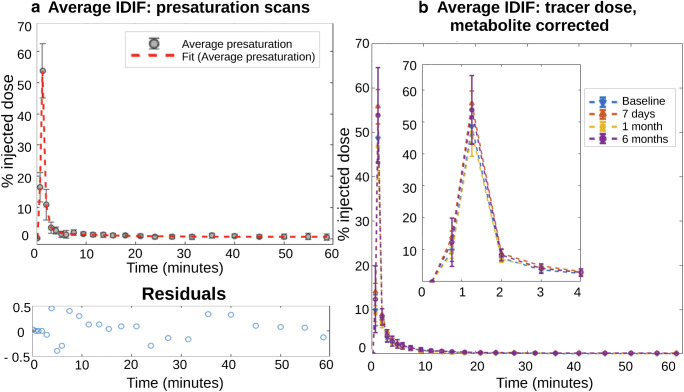
**a** Mean IDIF extracted from the presaturation studies, with fit and residuals underneath. **b** Mean and standard deviation of extracted IDIFs, fitted and metabolite corrected for the four time points with the peak inlaid

The left panel of Fig. 2a shows the average IDIF extracted from the presaturation experiments, with the curve fit and a plot of the residuals. The right panel (b) is the average IDIFs extracted for tracer experiments at each of the time points, which show the small difference between peak values (not significant). Peak magnitude of the IDIF correlates very well with the injected dose (all, Pearson *R* = 0.79; baseline, *R* = 0.93; 7 days, *R* = 0.95; 1 month, *R* = 0.80; and 6 months, *R* = 0.66 (0.84 excluding one outlier)).

### Parameter estimates

**Fig. 3:**
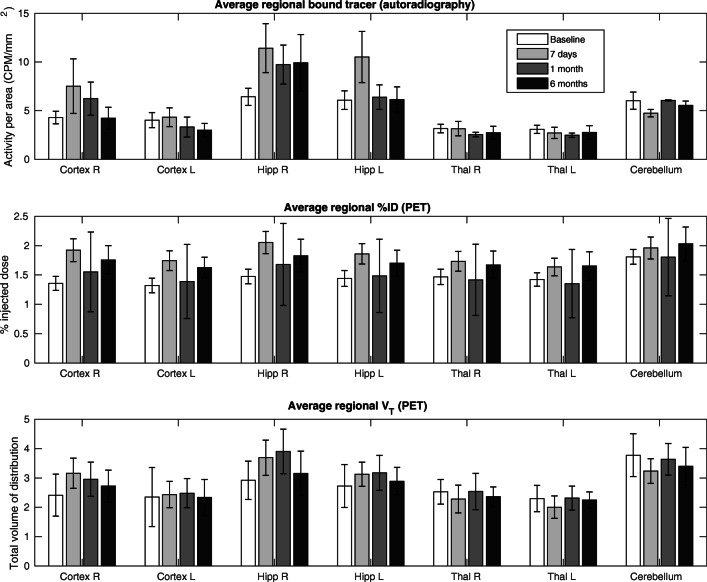
Regional parameter estimates for each time point post kainic acid injection (mean and standard deviation). Top, Autoradiography (cpm/mm^2^) (*n* = 3 at each time point); Middle, %ID without 4D-RRD; Bottom, PET V_T_ after 10 iterations of 4D-RRD

The plots in Fig. 3 show that the regional pattern of the V_T_ is similar to that of the autoradiography in comparison to the %ID which has a much flatter regional distribution. The similarity between the V_T_ values and autoradiography is strong within the cortex and the cerebellum, where the %ID is more variable over the 4 time points, showing an increase at 7 days and 6 months for all regions. There is a discrepancy between the peak at 7 days in the hippocampi seen in the autoradiography but not in the V_T_. This is likely due to the hippocampus definition within the mouse brain atlas being of much larger volume when compared to the ROI drawn on the autoradiography slices (see Supplementary Fig. 4).

To demonstrate the goodness of fit for the basis pursuit denoising and parameter estimation, regional TACs have been extracted from the original images and from images where only the denoising/parameter estimation has been applied, with no resolution recovery. The extracted TACs from 5 regions for two representative animals (one baseline and one at 1-month post KA) are shown in Supplementary Fig. 6, along with the residuals. The fits are visually quite good, and there is no clear pattern in the residuals, showing the number of basis functions used is appropriate. Because the fitting happens at a voxel-wise level in this process, an average voxel-wise map of the residuals are also shown in Supplementary Fig. 7 for each time point, showing that on average the fits stay within 3% of the curves.

**Fig. 4:**
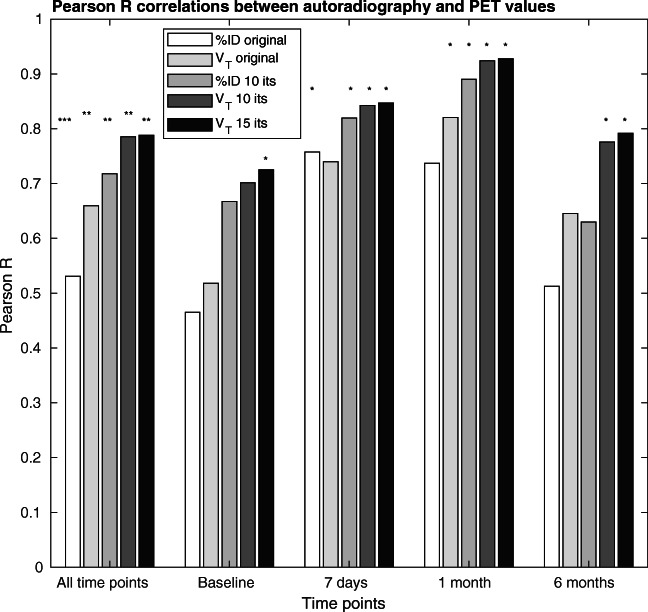
Pearson *R* coefficients between [^3^H]-DPA714 autoradiography and PET measures for each time point post kainic acid injection. The coefficients between the autoradiography and %ID or V_T_ are shown for original images and after 4D-RRD processing. The stars represent the significance level of the Pearson *R* coefficient (*p < 0.05, **p < 0.01, ***p < 0.001)

The correlations between the parameters from the PET scans and the autoradiography are shown in Fig. 4. For each time point, and over all animals at all time points, there is an increase in the correlation between the autoradiography and the PET parameter with increasing iteration, which plateaus around 10 iterations. Looking at the %ID for 0 and 10 iterations of 4D-RRD, the Pearson *R* increased by 35% by doing 4D-RRD only (from 0.53 to 0.72). Correlations are further improved using the V_T_ value instead of the %ID with a total increase of 48% (from 0.53 to 0.79). The V_T_ values without 4D-RRD (0 its) increased the correlation over all time points by 24% compared to %ID without 4D-RRD (from 0.66 to 0.79). The significance of the Pearson *R* changes depending on the method applied, giving a better indication of the strength of each method, as indicated as significance levels in Fig. 4. The Pearson *R* coefficient is significant for all methods used when including all time points at once (7 regions at 4 time points = 28 correlation points). Looking at the individual time points (7 regions = 7 correlation points each), without 4D-RRD, the %ID original method only gave significant Pearson *R* correlations at 7 days time point, and the V_T_ estimate without 4D-RRD was only significant at the 1-month time point. Applying 4D-RRD generally produced significant Pearson *R* coefficients, except for the Baseline group, where only the 4D-RRD with 15 iterations had a significant Pearson *R*.

As explained in the methods, the parameter estimate, V_T_ represents the V_F_ + V_NS_ + V_S_, whereas the autoradiography represents the specific binding only. ^18^F-DPA714 being a highly specific tracer, the non-specific binding should not be a cause of discrepancy. This is shown by the blocking study carried out on the autoradiography slides where the non-specific binding is negligible to non-existent (Supplementary Fig. 5).

### PET parametric maps: comparison with autoradiography

**Fig. 5:**
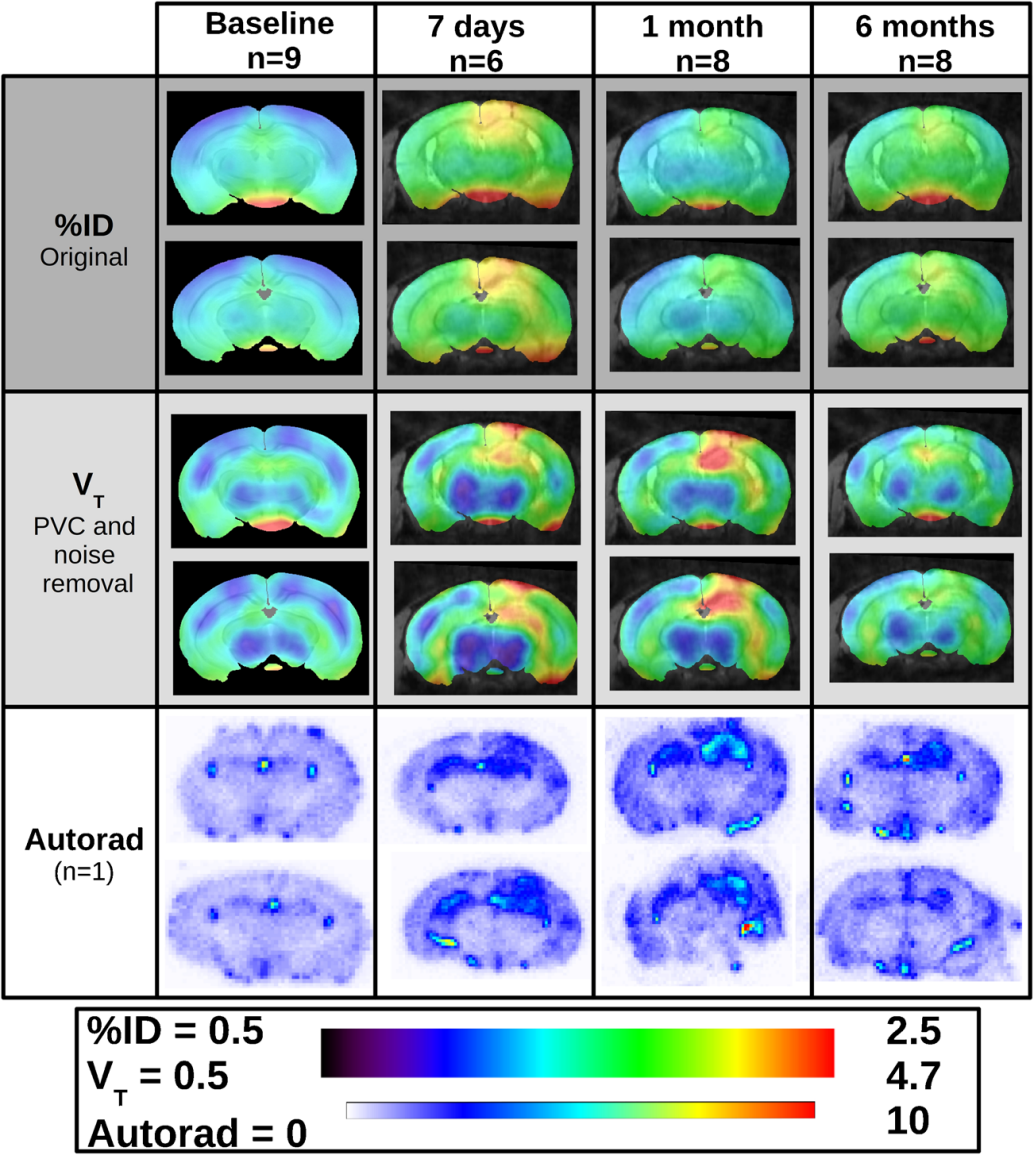
Average parametric maps for each time point post kainic acid (baseline, 7 days, 1 month and 6 months). The images are for %ID (dark grey segment) and V_T_ (light grey segment) at a ventral (top images) and dorsal (bottom images) hippocampal slice. The bottom segment (white) shows the [^3^H]DPA-714 autoradiography at the same time points for the same slices for one representative animal

Figure 5 shows the group averages for all mouse brain scans at each time point for the %ID on original images (top), the V_T_ after 10 iterations of 4D-RRD (middle) and one representative animal for each time point of autoradiography. Visually, the lesion is much clearer at each time point in the V_T_ maps compared to the %ID and resembles the distribution of tracer binding in the autoradiography images more closely. At 7 days, it is possible to make out sub-regions of the hippocampus with higher binding in the V_T_ map. The MRI overlaid with the PET images at all time points (except for baseline, where the atlas MRI was used) was the representative MRI obtained at 6 months post KA induction to show the morphology changes. This MRI image is also displayed without the PET parametric map in Supplementary Fig. 2.

## Discussion

The ability to extract an input function directly from the image will be useful in small animal studies where it is extremely challenging to do arterial sampling, and there is no true reference region available, such as in TSPO imaging. In this study, we developed and assessed a robust and reproducible method to extract the IDIF from dynamic mouse whole-body scans using a FA approach. This mouse-specific IDIF is used for a resolution recovery and denoising process that outputs an image with better spatial resolution and less noise, as well as a map of estimates of the V_T_. The V_T_ estimated was compared at a regional level to quantified ^3^H autoradiography across groups of animals in a mouse model of epilepsy.

The 4D-RRD method used in this study was proposed by Wimberley et al. [5] and Reilhac et al. [6] and uses an iterative deconvolution combined with a basis pursuit denoising that requires an input function or reference region TAC, neither of which are possible for TSPO imaging in the mouse brain. Extracting an IDIF using FA allows the use of this effective method to improve quantitative accuracy. The visual improvement obtained by using the 4D-RRD is evident in the parametric maps (Fig. 5), and the quantitative improvement is shown in the correlations (Fig. 4) where the 4D-RRD process improves the Pearson *R* between quantified autoradiography and in vivo PET measurements from *R* = 0.53 in the original %ID image to *R* = 0.79 in the V_T_ map of 10 iterations. Applying the basis pursuit method of voxel wise V_T_ without any resolution recovery (0 its) does improve the Pearson *R* (*R* = 0.66) compared to using %ID images without any resolution recovery (*R* = 0.53). When looking at the individual time points, the correlations improve when the pathology is the strongest and thus yields larger regional differences, and the methods used impact the significance of the Pearson correlation coefficients. With 7 regions included in the correlation, the only method that showed a significant correlation at all time points was using the V_T_ estimates from the 4D-RRD with 15 iterations. Using 10 iterations of the 4D-RRD also produced significant correlations for all time points, except baseline, where there is less regional variation. The improvement of the correlations is echoed when looking at the confidence intervals of each Pearson coefficient (Supplementary Table 1).

Separating the effect of the basis pursuit denoising and parameter estimation from the resolution recovery and presenting that alongside the combined techniques (4D-RRD) demonstrate that using both elements gives more accurate binding parameter estimates than just using one of the techniques alone. This echoes the results shown in Reilhac et al. [6] and Wimberley et al. [5] where the resolution recovery greatly improved the accuracy of the parameter estimates and the basis pursuit denoising reduced the variability.

FA has been previously used in medical image analysis for different purposes, including the extraction of a blood curve. In our application, the use of an injection pump with a tracer injection time of 1 min made the blood curve very reproducible, allowing for several physiological constraints to guide FA. In previous applications [21, 24], the only constraint applied was positivity, whereas in our application, we also apply a similarity constraint to a curve extracted from a presaturation experiment. In addition, the whole mouse in the PET FOV is used, allowing more pools of blood to be included in the analysis, which facilitates the identification of the IDIF. Thirdly, the normalization procedure is different from what has been previously described, using the activity in the whole field of view to normalize the factor curves. Lastly, this study estimated parameters of ^18^F-DPA-714 binding to TSPO and compared them to quantified autoradiography measurements.

There have been methods of IDIF extraction proposed that place a ROI within the aorta in order to extract a radioactivity signal from the blood [10]; however, this measurement gives the whole blood signal rather than the plasma. This is not ideal for TSPO scans as the protein is present on red blood cells and the signal from a ROI placed over a blood pool would be contaminated with blood-bound TSPO [38]. The TAC would also be contaminated by PVC effects of TSPO expression in the blood vessels and other nearby sources. One strong advantage of using FA to extract an IDIF for TSPO tracers is that the FA model accommodates mixed signal within voxels and can estimate the contribution of each component (e.g. bound tracer and free tracer) within voxels. To ensure that we were identifying the right signals with FA, a set of presaturation experiments was conducted, where all the TSPO in the mouse body was blocked. In presaturation experiments, the whole blood and plasma come into equilibrium very quickly, which is not reached at all during tracer dose experiments due to TSPO present within the blood [38]. Therefore, using the curve extracted from the presaturation experiments as a constraint for the FA of tracer dose experiments, we can separate the signal that comes from the specific binding within whole blood, and the IDIF extracted would then be closer to the shape of the plasma than the whole blood. Looking at Fig. 1, we have identified curves that resemble specific binding and plasma with respect to their spatial distribution as well as the curve shape.

Quantification methods that propose the use of a pseudo-reference region such as supervised clustering analysis (SVCA) [39] have been tested in rat brain studies but with varying success compared to application in the human due to the limited number of voxels within the rodent brain. The limited number of voxels is even more of a problem for the mouse brain. SVCA has been tested for use in the rat brain for ^18^F-DPA714 [40]; however, due to high noise levels, it was modified to be used at a region level as opposed to a voxel level, in effect identifying the region with the lowest TSPO binding to be taken as the pseudo-reference region. In pathologies where there are many brain regions with elevated TSPO expression or whole brain expression of TSPO, there will then be fewer voxels or regions from the already limited number available for the algorithm to select. In this case, the algorithm produces noisy or contaminated TACs to be a part of the pseudo-reference curve. This has recently been shown by one group [41] and would only be more present in the mouse brain due to the smaller size of the brain, and fewer voxels present. Additionally, the set-up of these methods require the definition of the ideal reference curve which is generally taken from the region with the lowest TSPO binding in the healthy brain, which will still have TSPO present. Extending beyond the brain to the whole body and concentrating on blood allow for larger pools of voxels to be included, and FA has the benefit of allowing voxels to be made up of a mixture of the factors identified which are related to underlying physiological signals.

The method presented in this article should be applicable for other tracers. TSPO is a particularly challenging case where a robust IDIF is very useful due to the ubiquitous nature of the protein where it is present in healthy brain tissue and on red blood cells. For quantification of TSPO scans, other commonly used methods are contaminated by the presence of the target within the blood, heart, lung and vessel tissue or the low level within the brain tissue.

One limitation of this study is the lack of arterial input function from the mice for validation purposes. We did not have access to the usual gold standard of comparison for our extracted IDIF (arterial blood sampling) due to the limited blood volume of the mouse. We instead used quantified autoradiography as a comparison for our parameter estimates. There can be differences between autoradiography and PET: in vivo vs ex vivo morphology changes, resolution differences and differences in region definition, which can explain the difference between autoradiography and V_T_ as seen in the hippocampus. Along with the morphology differences, discrepancies between the autoradiography and the PET parameter estimates could be related to the free tracer in the tissue, which could vary regionally, especially due to the disruption in the blood brain barrier caused by the pathology. The non-specific binding present in the PET parameter estimate could also play a role in any discrepancies, but Supplementary Fig. 5 shows there is negligible non-specific binding of ^18^F-DPA-714 in the mouse brain.

Additionally, not having access to the arterially sampled input function means that there is no means to have an individually sampled metabolite curve, so we used a population-based metabolite curve. A metabolite curve experiment would need to be repeated in an additional group of animals where metabolic differences are expected such as due to systemic disease state or pharmacological changes.

## Conclusion

We presented a FA approach to IDIF extraction based on whole-body mouse PET scans, and demonstrated that it is robust and can be used in resolution recovery and noise reduction methods and parameter estimation for longitudinal studies in the mouse when a reference region is not available. This approach is especially useful for longitudinal whole-body imaging studies in animals, where it is logistically or practically difficult to do arterial blood sampling.

## Electronic supplementary material

ESM 1 (DOCX 1458 kb)

## Data Availability

The data that support the findings of this study are available from the IMIV research group at the SHFJ-CEA, but restrictions apply to the availability of these data, which were used under licence for the current study, and so are not publicly available. Data are however available from the authors upon reasonable request and with permission of the IMIV research group at the SHFJ-CEA.
