# Supplementary material for: Longitudinal mouse-PET imaging: a reliable method for estimating binding parameters without a reference region or blood sampling
Source: Eur J Nucl Med Mol Imaging. 2020 Mar 24;47(11):2589–601. doi: 10.1007/s00259-020-04755-5 (PMC7515949; doi:10.1007/s00259-020-04755-5)
Supplement: Supplementary file 1 — (DOCX 1458 kb) [file 259_2020_4755_MOESM1_ESM.docx]

**Supplementary information:**

**Supplemental Figure 1:** Population based metabolite curve. Plasma samples were obtained from 12 healthy C57BL/6 mice (21.92 ± 1.68 g) which had received an i.v. injection of 0.52 ±.0.05 MBq/g of [^18^F]DPA-714 at 10, 20, 40 and 60 minutes before sacrifice. Blood was sampled by cardiac puncture under isofluorane 1% and centrifuged for 5 min at 4°C (3500 rpm) to obtain cell-free plasma. The total radioactivity in the plasma samples was measured using a cross-calibrated gamma counter (Cobra Quantum D5003, Perkin Elmer, Waltham, MA, USA). [^18^F]DPA-714 was quantified using a previously published tissue extraction SPE analysis method[34]. The radioactivity associated with [^18^F]DPA-714 was expressed as as a fraction of total plasma radioactivity applied on the SPE cartridge (i.e. sum of the radioactivity in each SPE fraction). Data were fitted by a nonlinear regression analysis using the following second order exponential equation (Origin Pro software version 8.5). The variability through the points (especially point 4 lying away from the curve) can be explained by: 1) The low amount of blood (hence low volume of plasma) we could collect due to the low blood volume of the mouse; 2) Due to the low blood volume with an excess of anticoagulant, some samples could have been hemolized, affecting the blood to plasma ratio; 3) The low number of animals per point.


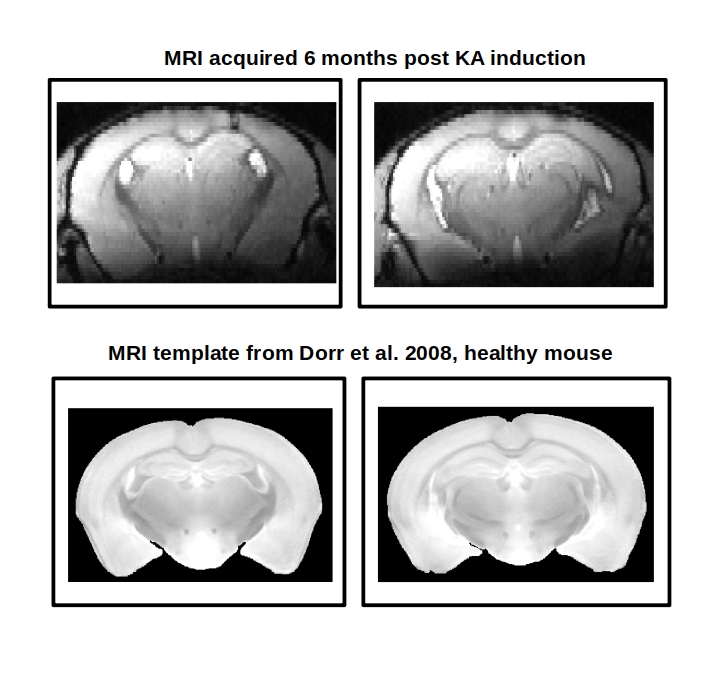


**Supplemental Figure 2:** MRI acquired at the 6 months time point (top row) showing morphological changes in the right hippocampus.


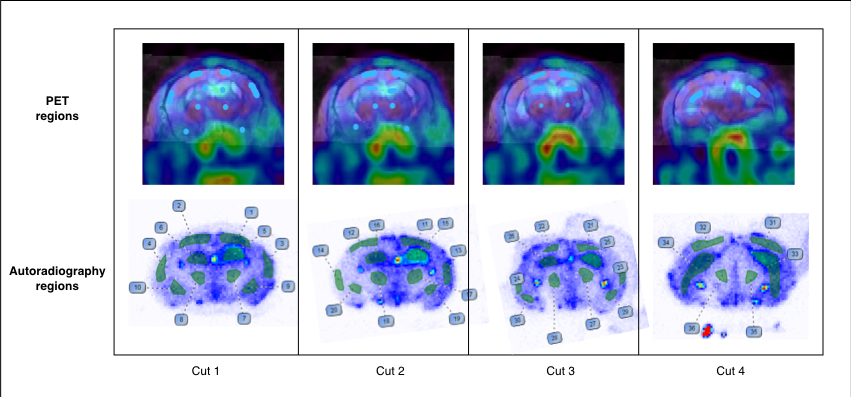


**Supplemental Figure 3:** ROI placement used for the autoradiography images to obtain a correlation with the regions in the Dorr et al. Atlas [29].


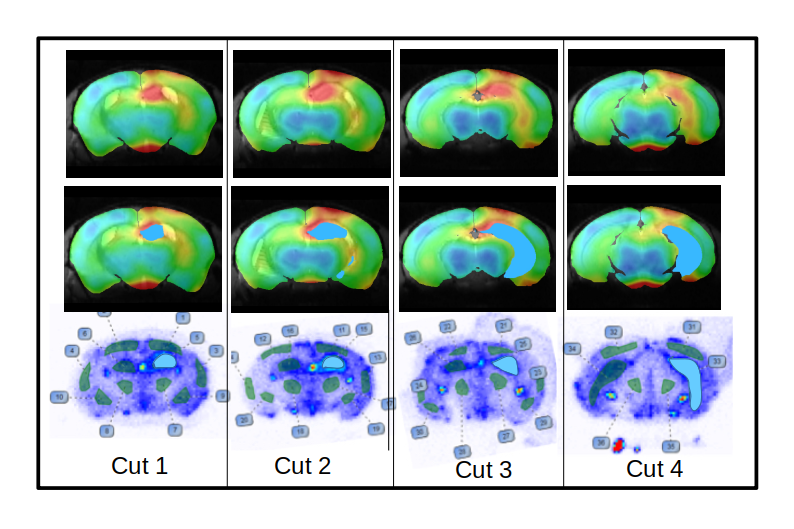


**Supplemental Figure 4:** ROI placement of hippocampus used for the PET images from the Dorr atlas compared to one example of a hippocampus ROI manually drawn on the autoradiography. The ROIs are shown in blue on both sets of images except the top row, which shows only the average of the VT maps at each time point. The hippocampus in the atlas is a larger volume with more brain tissue with lower activity, especially ventrally, due to a stricter definition of the hippocampus manually.


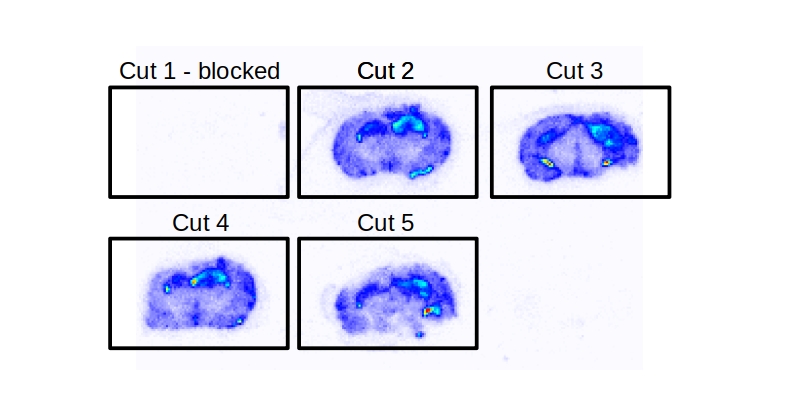


**Supplemental Figure 5:** ROI One set of slices from an animal (1 month time point after KA induction) demonstrating the blocking (using 11C-PK11195) for determining the non specific binding as described in the methods. As can be seen, there is very little to no non specific binding present, as DPA-714 is a high specificity for TSPO.


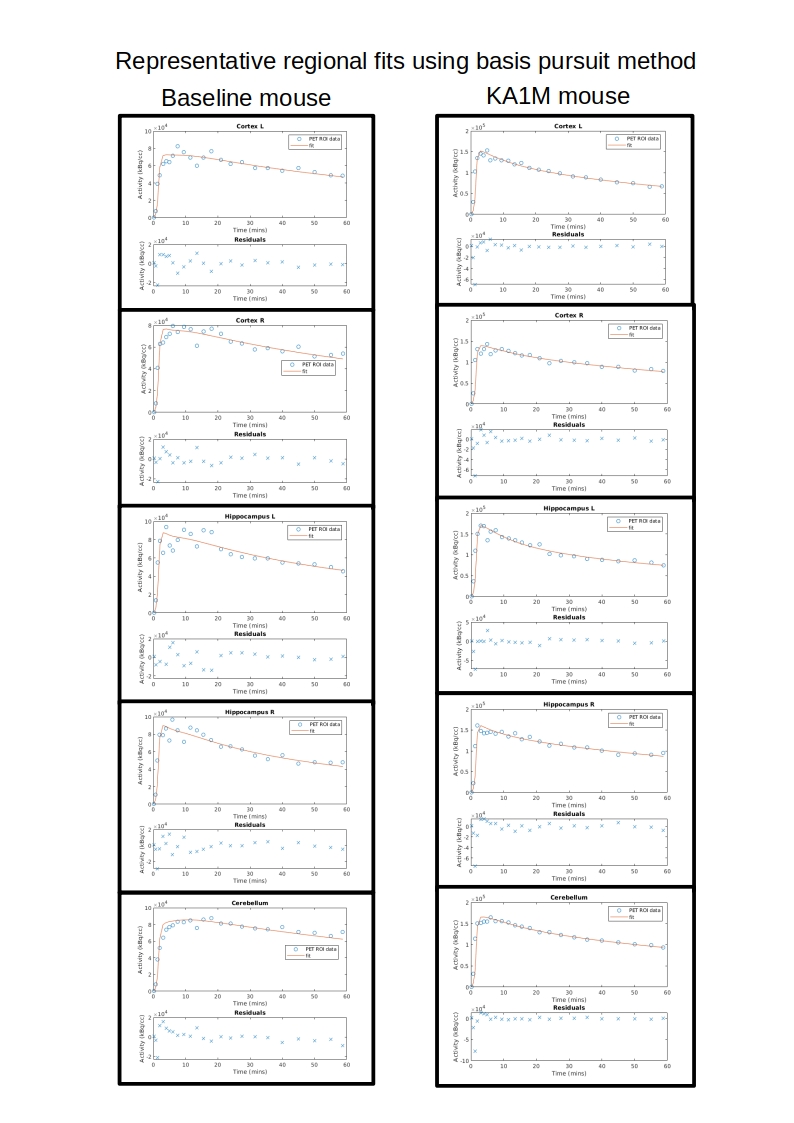


**Supplemental Figure 6** Representative regional TACs from the original images (round dots) and the fitted images using the basis pursuit method at the voxel wise level. TACs come from one baseline animal and one KA animal (one month post KA induction).


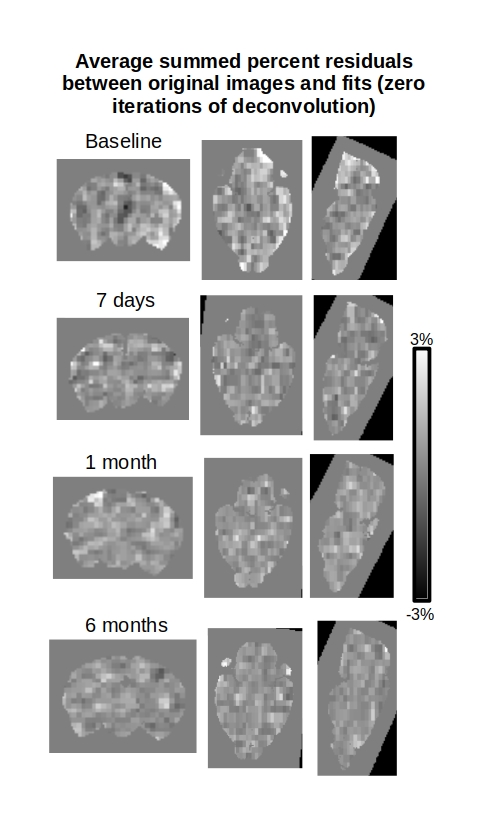


**Supplemental Figure 7:** Mean voxel wise residuals (average difference between original voxel TAC and fitted TAC) as a percentage of the original TAC. These percentages were averaged over all animals at each of the time points. The fitted TAC sum of residual lies within 3% of the original.

| All | R | p | Confidence interval low | Confidence interval high |
| --- | --- | --- | --- | --- |
| %ID original | 0.53 | 0.00 | 0.20 | 0.75 |
| VT original | 0.66 | 0.00 | 0.38 | 0.83 |
| %ID 10 its | 0.72 | 0.00 | 0.47 | 0.86 |
| VT 10its | 0.79 | 0.00 | 0.58 | 0.90 |
| VT 15its | 0.79 | 0.00 | 0.59 | 0.90 |
| Baseline | R | p | Confidence interval low | Confidence interval high |
| %ID original | 0.47 | 0.29 | -0.44 | 0.90 |
| VT original | 0.52 | 0.23 | -0.39 | 0.91 |
| %ID 10 its | 0.67 | 0.10 | -0.17 | 0.95 |
| VT 10its | 0.70 | 0.08 | -0.11 | 0.95 |
| VT 15its | 0.76 | 0.05 | 0.00 | 0.96 |
| 7 days | R | p | Confidence interval low | Confidence interval high |
| %ID original | 0.76 | 0.05 | 0.01 | 0.96 |
| VT original | 0.74 | 0.06 | -0.03 | 0.96 |
| %ID 10 its | 0.82 | 0.02 | 0.17 | 0.97 |
| VT 10its | 0.84 | 0.02 | 0.24 | 0.98 |
| VT 15its | 0.85 | 0.02 | 0.26 | 0.98 |
| 1 month | R | p | Confidence interval low | Confidence interval high |
| %ID original | 0.74 | 0.06 | -0.04 | 0.96 |
| VT original | 0.82 | 0.02 | 0.18 | 0.97 |
| %ID 10 its | 0.89 | 0.01 | 0.42 | 0.98 |
| VT 10its | 0.92 | 0.00 | 0.56 | 0.99 |
| VT 15its | 0.93 | 0.00 | 0.58 | 0.99 |
| 6 months | R | p | Confidence interval low | Confidence interval high |
| %ID original | 0.51 | 0.24 | -0.39 | 0.91 |
| VT original | 0.65 | 0.12 | -0.21 | 0.94 |
| %ID 10 its | 0.63 | 0.13 | -0.23 | 0.94 |
| VT 10its | 0.78 | 0.04 | 0.05 | 0.97 |
| VT 15its | 0.79 | 0.03 | 0.10 | 0.97 |

**Supplemental Table 1:** Pearson coefficients from Figure 4 with the associated significance value (p) and the confidence intervals, top and bottom.
